# Extreme obesity is a strong predictor for in-hospital mortality and the prevalence of long-COVID in severe COVID-19 patients with acute respiratory distress syndrome

**DOI:** 10.1038/s41598-022-22107-1

**Published:** 2022-11-01

**Authors:** Lars Heubner, Paul Leon Petrick, Andreas Güldner, Lea Bartels, Maximillian Ragaller, Martin Mirus, Axel Rand, Oliver Tiebel, Jan Beyer-Westendorf, Martin Rößler, Jochen Schmitt, Thea Koch, Peter Markus Spieth

**Affiliations:** 1 Department of Anesthesiology and Intensive Care Medicine, University Hospital “Carl Gustav Carus”, Technische Universität Dresden, Dresden, Germany; 2 Institute of Clinical Chemistry, University Hospital “Carl Gustav Carus”, Technische Universität Dresden, Dresden, Germany; 3 Division of Hematology and Hemostasis, Department of Medicine I Thrombosis Research, University Hospital “Carl Gustav Carus”, Technische Universität Dresden, Dresden, Germany; 4 Center for Evidence-Based Healthcare (ZEGV), University Hospital “Carl Gustav Carus” and “Carl Gustav Carus” Faculty of Medicine, Technische Universität Dresden, Dresden, Germany

**Keywords:** Medical research, Respiratory distress syndrome

## Abstract

Acute Respiratory Distress Syndrome (ARDS) is common in COVID-19 patients and is associated with high mortality. The aim of this observational study was to describe patients’ characteristics and outcome, identifying potential risk factors for in-hospital mortality and for developing Long-COVID symptoms. This retrospective study included all patients with COVID-19 associated ARDS (cARDS) in the period from March 2020 to March 2021 who were invasively ventilated at the intensive care unit (ICU) of the University Hospital Dresden, Germany. Between October 2021 and December 2021 patients discharged alive (at minimum 6 months after hospital discharge—midterm survival) were contacted and interviewed about persistent symptoms possibly associated with COVID-19 as well as the quality of their lives using the EQ-5D-5L-questionnaire. Long-COVID was defined as the occurrence of one of the symptoms at least 6 months after discharge. Risk factors for mortality were assessed with Cox regression models and risk factors for developing Long-COVID symptoms by using relative risk (RR) regression. 184 Patients were included in this study (male: n = 134 (73%), median age 67 (range 25–92). All patients were diagnosed with ARDS according to the Berlin Definition. 89% of patients (n = 164) had severe ARDS (Horovitz-index < 100 mmHg). In 27% (n = 49) extracorporeal membrane oxygenation was necessary to maintain gas exchange. The median length of in-hospital stay was 19 days (range 1–60). ICU mortality was 51%, hospital mortality 59%. Midterm survival (median 11 months) was 83% (n = 55) and 78% (n = 43) of these patients presented Long-COVID symptoms with fatigue as the most common symptom (70%). Extreme obesity (BMI > 40 kg/m^2^) was the strongest predictor for in-hospital mortality (hazard ratio: 3.147, confidence interval 1.000–9.897) and for developing Long-COVID symptoms (RR 1.61, confidence interval 1.26–2.06). In-hospital mortality in severe cARDS patients was high, but > 80% of patients discharged alive survived the midterm observation period. Nonetheless, most patients developed Long-COVID symptoms. Extreme obesity with BMI > 40 kg/m^2^ was identified as independent risk factor for in-hospital mortality and for developing Long-COVID symptoms.

**Trial registration** DRKS-ID DRKS00027856.

## Introduction

According to WHO statistics, more than 500 million people globally were infected by SARS-CoV-2 and approximately up to 6 million people died by or with COVID-19^1^. COVID-19 can cause severe acute respiratory distress syndrome (ARDS) with the need of mechanical ventilation (MV), and, for more severe cases, inhaled nitric oxide^2^ and extracorporeal membrane oxygenation (ECMO)^3^ are used as rescue therapies. The importance of ECMO therapy in SARS-CoV-2 ARDS is highlighted by a remarkable increase in the number of applications^4^. From the beginning in March 2020 till May 2021 the amount of ECMO applications in Europe raised from 68 to 4337^4^.

Besides respiratory support, various pharmacological interventions for SARS-CoV-2 ARDS were tested—in particular during the early stage of the pandemic. Despite these efforts, ICU mortality remained high ranging from 40 to > 80%^5–7^. In addition to the infection and inflammatory damage to lung tissue, various mechanisms of hypercoagulopathy and fibrinolytic disorders have been described in patients infected by SARS-CoV-2^8–21^ leading to high incidences of deep vein thrombosis and pulmonary embolism^8,18,22,23^. Compared to other types of ARDS, venous thromboembolism (VTE) rates of 20–58%^8,18,22–24^ are extremely high. Recent studies implicated a close connection between the occurrence of thromboembolic events and patients outcome^17^. As a consequence, strict anticoagulation recommendations were issued^12,23^ from the early stages of the pandemic. However, data on the optimal dosing of anticoagulant therapy are conflicting^25–28^ and the methodology of randomized trials addressing this topic suffered from major limitations and confounders.

Hyperinflammation or cytokine storm is often described as a common feature with high impact on COVID-19 morbidity and mortality^29^. Several pharmaceutical treatments were tested to prevent or treat hyperinflammation. Since the RECOVERY trial was published in July 2020—showing lower 28-day mortality in hospitalized COVID-19 patients with administration of dexamethasone^30^—institutional guidelines changed including glucocorticoid administration in all COVID-19 ARDS patients. Furthermore, later studies showed that, among critical ill COVID-19 patients the use of tocilizumab—a humanized monoclonal antibody against interleukin-6—is associated with lower in-hospital mortality^31^.

Finally, even for patients surviving the acute phase of severe SARS-CoV-2 infections or SARS-CoV-2 ARDS, increasing evidence suggests long-term sequelae for a large proportion of patients.

The term “Long-COVID” was first mentioned in May 2020 by Elisa Perego, who was experiencing prolonged symptoms after an infection with SARS-CoV-2^32^. Based on the NICE-guideline, published in December 2020^33^, Long-COVID is defined as newly occurring symptoms which were either not present during the acute phase of infection or persisted for longer than 4 weeks. In contrast, post-COVID should be considered when ongoing symptoms persist 3 months post-infection. In December 2021 the WHO Clinical Case Definition Working Group published a definition for post-COVID following a Delphi consensus. The five groups discussing the definition consisted of 61 patients, 18 patient-researchers, 138 external experts, 33 WHO staff, and 15 others. Items were evaluated using a nine-point Linkert scale. Items with a low rating in round one were later removed, while new items suggested by participants were added. The participants defined post-COVID as occurring “usually three months from the onset of COVID-19 with symptoms that last for at least two months and cannot be explained by an alternative diagnosis. Common symptoms include fatigue, shortness of breath, and cognitive dysfunction […] and generally have an impact on everyday functioning”. There was no differentiation between persisting and newly occurring symptoms^34^.

The aim of this observational study was to describe characteristics and outcome of cARDS patients, discussing the role of potential risk factors for in-hospital mortality in these patients. Furthermore, patients discharged alive were evaluated for survival after minimum of 8 months—defined as midterm survival—and the prevalence of Long-COVID symptoms.

## Methods

### Study design

This was a single-center, retrospective observational study performed in a tertiary German university hospital specialized in lung diseases (University Hospital “Carl Gustav Carus” of Technical University of Dresden). All patients admitted to University hospital “Carl Gustav Carus” Dresden with polymerase chain reaction confirmed COVID-19 infection presenting with severe respiratory failure according to ARDS criteria^35^ (Horovitz-index < 300mHg), requiring invasive mechanical ventilation between March 2020 and March 2021 were enrolled in this study and mid-term outcome and the prevalence of Long-COVID were assessed by follow up > 6 months post discharge.

### Data collection and outcome definitions

All patients’ data were recorded during the entire ICU stay. Primary outcome was defined as mortality during hospital stay. Secondary outcome was defined as occurrence of Long-COVID symptoms.

Sepsis was defined according to the International Consensus Definitions for Sepsis and Septic Shock (Sepsis-3)^36^, additional septic shock was defined as persistent hypotension with the need of catecholamine drugs to maintain mean arterial pressure ≥ 65 mmHg despite adequate volume substitution—and Serum lactate value > 2 mmol/l^36,37^. SOFA score and Charlson Comorbidity Index (CCI) score were calculated using standardized protocols at day of ICU admission.

All patients in our ICU were treated according to the same standard operating procedure (SOP) for anticoagulation therapy with consulting support by the department of internal medicine to identify patients at high risk for thrombosis at the time of ICU admission. On ICU admission, all patients were screened for venous thromboembolism (VTE) using complete compression ultrasound (cCUS) SOPs. Preexisting PE was detected by thoracic computed tomography pulmonary angiography (CTPA). Additional cCUS and CTPA were performed, if any clinical signs of venous or arterial thrombosis or embolism occurred. If PE was diagnosed, following cCUS was performed in every single case. Patients without venous or arterial thromboembolism received standard weight-based sub-therapeutic unfractionated heparin (target aPTT of 40–50 s) or intermediate doses of low molecular weight heparin (100 aXa units/kg/day). All patients with confirmed ATE/VTE received therapeutic weight-based unfractionated heparin (target aPTT of 60–80 s) or low molecular weight heparin (200 aXa units/kg/day). Patients with contraindications for full therapeutic anticoagulation received a patient specific therapy, according to benefit-risk assessments which included thrombus burden, bleeding risk or current bleeding intensity. Anticoagulant treatment target ranges for such patients were aPTT 50–60 s or LMWH dosages between 100 and 200 units/kg/day. Patients suffering from heparin-induced-thrombocytopenia (HIT) were treated with direct thrombin inhibitors according to guidelines.

All patients with refractory severe hypoxemia fulfilling the EOLIA criteria^38^ were screened for necessity of extracorporeal membrane oxygenation (ECMO). Individual decision was taken in multidisciplinary deliberation process. ECMO was performed as femoro-jugular veno-venous bypass using percutaneous ultrasound guided insertion of drainage and return cannula.

### Laboratory analysis

Standard laboratory analyses including relative prothrombin time (PT in % of normal and INR), activated partial thromboplastin time (aPTT), fibrinogen, fibrin monomers and D-dimers on STA R Max3-Analyzers (STAGO Deutschland GmbH, Düsseldorf, Germany). PF 1 + 2 was analyzed applying LOCI-technology on an Atellica COAG 360 System (Siemens Healthcare GmbH, Erlangen, Germany).

Additional blood count analyses were performed using EDTA-tubes for hemoglobin concentration, white blood cell count and platelet count. A serum collecting tube was used for measurements of inflammatory parameters (CRP, Interleukin 2 and 6 (IL-2, IL-6) and Procalcitonin (PCT) and organ function monitoring (creatinine, bilirubin, and albumin)).

Every patient underwent VET and blood drawing for the laboratory analyses at the same time point each. Blood was drawn at least once daily for laboratory analysis. Laboratory parameters included into cox regression analysis for in-hospital mortality were selected due to clinical relevance and observations. Therefore, only values of d-dimers at admission to our ICU were included in regression analysis. Additional, maximum values of leucocytes, interleukin-6, procalcitonin, CRP, platelets as well as minimum values of platelets were included in further regression analysis. Thresholds were set according to clinical estimations.

### Assessment of long-COVID and Questionnaires

Between October 2021 and December 2021, all patients who consented to participate in the study were telephone-interviewed by a trained medical student with standardized questionnaires investigating specific persistent symptoms possibly associated with COVID-19 and the quality of their lives. The minimum interval between discharge and follow-up was defined as 6 months and varied between the patients. The questionnaires contain self-reported symptoms including fatigue, weakness, shortness of breath, cough, headache, and muscle or limb pain, smell disorder, sleep disorder, loss of hair, anxiety disorder or other neurological disorders. Furthermore, a standardized five-dimension five-level (EQ-5D-5L) questionnaire, and the EuroQol Visual Analogue Scale (EQ-VAS) was used to analyze quality of life. Participants were questioned to report symptoms (persistent or newly occurring) different than before COVID-19 at the time of the interview. The EuroQol is a validated questionnaire with two components. EQ-5D-5L, is a health state classification system with five different dimensions: mobility, self-care, usual activities, pain or discomfort, and anxiety or depression. Each dimension has to be rated ranging from 1—“no problems” to 5—“unable to/extreme problems” to classify severity of symptoms. The EQ-VAS is the individual self-assessment of overall health ranging from 0 to 100 considered as “the worst health you can imagine” to “the best health you can imagine”. Furthermore, participants were asked if they could return to work and if permanent oxygen support and renal replacement therapy is necessary. Long-COVID was defined as the occurrence of one of the self-reported symptoms occurring at least 6 months after discharge, in accordance with German Guidelines for diagnostic of Long-COVID syndrome^39^.

### Statistical analyses

Statistical analyses were performed using the SPSS Statistics 27 software (IBM, Inc, Armonk, NY, U.S.) and R version 3.2.4. All categorical variables are described as absolute and relative frequencies; comparison between groups was done using Fisher's exact test. Continuous variables were presented as median and interquartile range (IQR 1st–3rd), group comparison was based on the Mann–Whitney U test. Cox regression analysis were performed to identify risk factors for mortality. In case of binary outcomes, we used robust Poisson regression^40^ for derivation of adjusted relative risks. Variables included in regression analysis were selected due to clinical estimations based on preexisting studies for ARDS (Tables 7 and 8). The Kaplan–Meier curves were constructed using R version 3.2.4 and group comparison were made using the log-rank test. The precision of relative risk (RR) estimates was quantified using 95%-confidence intervals (CIs). Significance level was set at 0.05.

### Ethics

The study was performed in accordance with the Declaration of Helsinki. The study protocol was approved by the Ethics Committee from of the Technical University Dresden, Germany (BO-EK-374072021) and registered at the German Clinical Trials Registry (DRKS0027856). According to german law, informed consent was not required due to the retrospective and observational design of the study.

## Results

### Short-term outcome

#### Characteristics of the cohort

**Figure 1:**
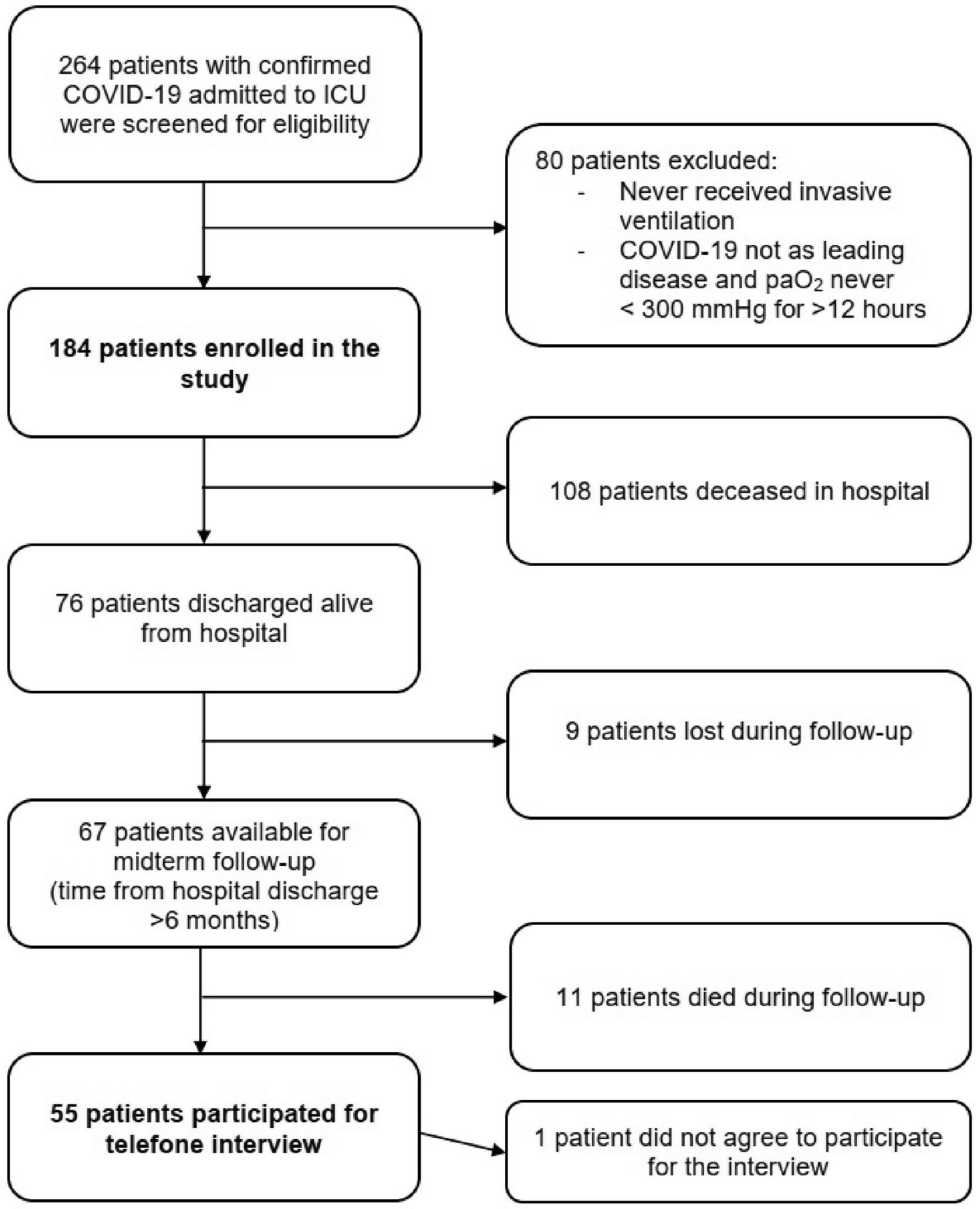
Flow of patient screening and enrollment. *ICU* intensive care unit, *ARDS* acute respiratory distress syndrome, *paO*_*2*_ arterial oxygen partial pressure.

Flow of patients screening and enrollment is shown in Fig. 1. Between 03/2020 and 03/2021, 184 patients were treated for severe respiratory failure secondary to COVID-19 in our ICU and were included in this study. Median age was 67 years (range 25–92, IQR 61–73) and 73% of the patients were men (n = 134). All patients showed critical organ failure on the day of study enclosure with a median SOFA score of 12 points (range 4–19, IQR 10–13).

All patients were intubated and mechanically ventilated, with a median Horovitz-index at hospital admission of 130 (range 45–450, IQR 82.5–150). Patients without ECMO (n = 135) had a lowest daily median Horovitz-index of 60 mmHg (range 23–225, IQR 52.5–75.0) during ICU stay. Patients were treated in a prone position in 61% (n = 113) at minimum of 16 h/d, median rate was 4 cycles (range 1–14, IQR 2–6).

In 34% (n = 62) additional inhaled nitric oxide therapy was needed and in 27% (n = 49) veno-venous ECMO was necessary to maintain gas exchange. Continuous veno-venous hemodialysis (CVVH) was necessary in 34% (n = 67). Corticoid therapy was applied in 90% (n = 165) during ICU stay. 4 patients (2%) received Immunoglobulins, CytoSorb® therapy was used in 8 (4%), in 19% reconvalescence plasma therapy (n = 34) was used and 20 patients (11%) received remdesivir (Table 1).

**Table 1:** ICU baseline characteristics during ICU stay.

	All patients	Range
n	184	
Intubated at ICU admission	133 (72.3%)	
ARDS mild at ICU admission	14 (7.6%)	
ARDS moderate at ICU admission	85 (46.2%)	
ARDS severe at ICU admission	82 (44.6%)	
Septic shock at ICU admission	24 (13.1%)	
First Horovitz-index at ICU	108.8 (82.5; 150)	45.0–450.0
Lowest Horovitz-index at ICU	60.0 (52.5; 75.0)	22.5–225.0
P_mean_ at admission [mbar]	20 (17; 22)	7–30
PEEP at admission [mbar]	14 (12; 15)	6–20
pH at admission	7.38 (7.33; 7.44)	6.81–7.62
PaCO_2_ at admission [kPa]	6.42 (5.64; 7.17)	3.23–15.90
SpO_2_ at admission [%]	93 (90; 96)	56–100
SOFA score at ICU admission	12 (10; 13)	4–19
D-dimers at ICU admission [ng/ml]	5178 (2326; 8936)	484–20,000
Lactate at ICU admission [mmol/l]	1.20 (0.90; 1.70)	0.40–9.90
Duration mechanical ventilation ICU [days]	12 (7; 17)	1–61
Reintubation	4 (2.2%)	
Prone position	113 (61.4%)	
Cycles of prone position	4 (2; 6)	1–14
Tracheostomy	82 (44.6%)	
Days from intubation to tracheostomy	12 (9; 15)	3–26
CRRT	67 (36.4%)	
Duration CRRT [h]	154.66 (31.51; 310.66)	1.44–906.53
ECMO	49 (26.6%)	
Duration ECMO [h]	274.66 (178.78; 353.04)	16.78–1068.31
Cytosorb	8 (4.3%)	
Duration cytosorb [h]	20.00 (17.17; 21.15)	8.67–51.00
Red cell transfusion	6 (2; 12)	1–40
NO inhalation	62 (33.7%)	
Corticosteroid	165 (89.7%)	
Immunoglobulin	4 (2.2%)	
Convalescent plasma	34 (18.5%)	
Remdesivir	20 (10.9%)	
Anticoagulation	184 (100%)	
Argatroban at any time on ICU	15 (8.2%)	
UFH at any time on ICU	140 (76.1%)	
LMWH at any time on ICU	115 (62.5%)	
Bacteremia	92 (50%)	
Staph. aureus bacteremia	12 (6.5%)	
Catheter associated bacteremia	24 (13%)	
Antibiotics	176 (95.7%)	
Antimycotics	28 (15.2%)	
CRP maximum value [mg/l]	261.2 (189.9; 342.1)	31.4–618.0
Interleukin 6 maximum value [pg/mL]	359.5 (123.0; 755.5)	8.6–792,732.0
Leucocytes maximum value [GPt/L]	19.06 (13.96; 25.91)	3.14–63.87
Leucocytes minimum value [GPt/L]	7.22 (4.89; 9.46)	0.20–22.47
Procalcitonin [ng/ml]	2.95 (0.91; 10.80)	0.09–373.20
Prothrombin fragment F1 + 2 [pmol/l]	468 (272; 930)	73–4948
Platelets maximum value [GPt/L]	315 (251; 418)	48–989
Platelets minimum value [GPt/L]	124 (73; 198)	1–469

Data are median (Interquartile range) or n (%).

*ICU* Intensive care unit, *ARDS* Acute respiratory distress syndrome, *P*_*mean*_ Mean pressure, *PEEP* Positive end-expiratory pressure, *PaCO*_*2*_ partial pressure of carbon dioxide, *SpO*_*2*_ Oxygen saturation, *SOFA* Sequential organ failure assessment, *CRRT* Continuous renal replacement therapy, *NO* Nitric oxide, *UFH* Unfractionated heparin, *LMWH* Low-molecular-weight heparin, *Staph.* Staphylococcus, *CRP* C-reactive protein, *ECMO* extracorporeal membrane oxygenation.

Duration between onset of symptoms and hospital admission was 5 days (range 0–23, IQR 0–7), for ICU admission 11 days (range 0–35, IQR 5–15) and for ECMO therapy 15 days (range 0–31, IQR 11–23). The majority of the patients had previous disease (97%, n = 179) with median Charlson Comorbidity Index of 3 points (range 0–12, IQR 2–5), while arterial hypertension (71%, n = 131), diabetes (43%, n = 79) and cardiovascular disease (25%, n = 45) were frequent and obesity was common in this cohort (median BMI 29, range 19–70, IQR 26–34). 14% (n = 26) presented obesity grade II (BMI 35–39.9 kg/m^2^) and 7% (n = 13) were noticed with severe obesity grade III (BMI ≥ 40 kg/m^2^) according to the WHO definition. Long-term drug intake was recorded frequently, mostly antihypertensive drugs were used in 52% (n = 95) of cases, beta blockers in 40% (n = 74), anti-platelet agents in 27% (n = 49) and oral anticoagulant drugs in 16% (n = 30). Only 7% (n = 12) were smokers (Table 2).

**Table 2:** Demographic and baseline characteristics of all patients on admission to our ICU.

	All patients	Range
n	184	
Male	134 (72.8%)	
Age [years]	67 (61; 73)	25–92
Body-Mass-Index [kg/m^2^]	29.22 (26.04; 33.60)	18.94–70.31
Time from first symptom to hospital admission [days]	5 (0; 7)	0–23
Time from first symptom to admission to our ICU [days]	11 (5; 15)	0–35
Time from first symptom to ECMO therapy [days]	15 (11; 23)	0–31
Direct transfer to our ICU from other hospital	121 (65.8%)	
External tracheostomy	14 (7.6%)	
External intubation	133 (72.3%)	
Invasive mechanical ventilation before admission to our ICU [days|	2 (0; 7)	0–20
NIV before admission to our ICU [days|	2 (1; 4)	1–22
Charlson ComorbidityIndex	3 (2; 5)	0–12
Arterial Hypertension	131 (71.2%)	
Cardiovascular disease	45 (24.5%)	
Neurovascular symptoms	18 (9.8%)	
Coronary artery disease	31 (16.8%)	
Thrombembolic events in medical history	11 (6.0%)	
Chronic arrhythmias	37 (20.1%)	
COPD	13 (7.1%)	
Other pulmonary disease	11 (6.0%)	
Nicotine abuse	12 (6.5%)	
Diabetes mellitus	79 (42.9%)	
Previous organ or bone marrow transplantation	9 (4.9%)	
Chronic renal failure	28 (15.2%)	
Chronic need of renal replacement therapy	8 (4.3%)	
Admission with trauma	8 (4.3%)	
ACE inhibitors	14 (7.6%)	
AT2 receptor blocker	85 (46.2%)	
Beta blocker	82 (44.6%)	
Antithrombotic drug	49 (26.8%)	
DOAC	30 (16.4%)	
Corticosteroids	21 (11.5%)	
Immunosuppressive drugs	10 (5.5%)	
Nosocomial infection	19 (10.3%)	

Data are median (Interquartile range) or n (%).

*ICU* Intensive care unit, *ECMO* extracorporeal membrane oxygenation, *NIV* non-invasive ventilation, *COPD* chronic obstructive pulmonary disease, *ACE* angiotensin-converting enzyme, *AT2* Angiotensin II, *DOAC* Direct oral anticoagulants.

#### Short-term survival and thromboembolic complications

Median in-hospital stay was 19 days (range 1–60, 14; 28) and end-of-treatment follow-up was 100% complete. 90 of 184 patients (49%) could be discharged alive from the anesthesiology ICU. 32 patients (17.4%) could be discharged to rehabilitation and the other alive patients were transferred to another ICU (n = 38; 20.7%) or to regular ward (n = 11; 6.7%) within the clinic (Table 3). Overall hospital mortality was 59% (n = 108). Non-survivors were at median 68 years (IQR 63–75) and significantly older than survivors (median 64 years, IQR 58–70, Table 4).

**Table 3:** Patients outcome all.

	All patients	Range
n	184	
Duration of hospital stay [days]	19 (14; 28)	
Duration of ANE-ICU stay [days]	13 (8.5; 19)	
Duration of stay at UKD [days]	17 (12; 24.5)	
VTE during ICU stay	85 (46.2%)	
DVT	58 (31.5%)	
Catheter associated thrombosis	5 (2.7%)	
PE	57 (31.0%)	
ATE	11 (6.0%)	
VTE before ICU admission	17 (9.2%)	
Pneumothorax	22 (12.0%)	
Lung emphysema	9 (4.9%)	
Mediastinal emphysema	13 (7.1%)	
Subcutaneous emphysema	17 (9.3%)	
Pleural effusion	81 (44.3%)	
Fusion in lung	15 (8.2%)	
**Status on day of discharge**		
Death	95 (51.6%)	
Regular ward	11 (6.0%)	
Other ICU	38 (20.7%)	
Rehabilitation clinic	32 (17.4%)	
Other hospital	8 (4.3%)	
Withdraw of care by patients will	105 (57.1%)	
Hospital survival	76 (41.3%)	
ICU survival	77 (41.8%)	
ANE-ICU survival	90 (48.9%)	

Data are median (Interquartile range) or n (%).

*ICU* Intensive care unit, *ANE-ICU* Intensive care unit of the Department of Anesthesiology and Critical Care Medicine, *UKD* University hospital Dresden, *DVT* Deep vein thrombosis, *VTE* Thromboembolic complications, *PE* Pulmonary embolism.

**Table 4:** Patients characteristics survival.

	Survivors	Range	Non-survivors	Range	*p*
n	90		94		
Male	60 (66.7%)		74 (78.7%)		
Age [years]	64 (58;70)	25–83	68 (63; 75)	33–92	**< 0.05**
Body-Mass-Index [kg/m^2^]	30.45 (26.12; 34.26)	20.81–52.47	27.78 (25.48; 33.14)	18.94–70.31	
Time from first symptom to hospital admission [days]	5 (0; 7)	0–50	4.5 (0; 8)	0–23	
Time from first symptom to admission to our ICU [days]	10 (5; 14)	0–28	11 (5; 16)	0–35	
Time from first symptom to ECMO therapy [days]	16 (13; 22)	4–25	15 (11; 23)	0–31	
Direct transfer to our ICU from other hospital	55 (61.1%)		66 (70.2%)		
External tracheostomy	6 (6.7%)		8 (8.5%)		
External intubation	60 (66.7%)		73 (77.7%)		
Invasive mechanical ventilation before admission to our ICU [days]	2 (0; 5)	0–20	3 (0; 7)	0–16	
Non-invasive mechanical ventilation before admission to our ICU [days]	2 (1; 3)	1–18	2 (1; 4)	1–22	
Charlson ComorbidityIndex	3 (2; 5)	0–11	3 (2; 6)	0–12	
Arterial Hypertension	66 (73.3%)		65 (69.1%)		
Cardiovascular disease	21 (23.3%)		24 (25.5%)		
Neurovascular symptoms	9 (10%)		9 (9.6%)		
Coronary artery disease	15 (16.7%)		16 (17.0%)		
Thromboembolic events in medical history	4 (4.4%)		7 (7.4%)		
Chronic arrhythmias	14 (15.6%)		23 (24.5%)		
COPD	8 (8.9%)		5 (5.3%)		
Other pulmonary disease	3 (3.3%)		8 (8.5%)		
Nicotine abuse	8 (8.9%)		4 (4.3%)		
Diabetes mellitus	42 (46.7%)		37 (39.4%)		
Previous organ or bone marrow transplantation	4 (4.4%)		5 (5.3%)		
Chronic renal failure	12 (13.3%)		16 (17.0%)		
Chronic need of renal replacement therapy	1 (1.1%)		7 (7.4%)		
Admission with trauma	6 (6.7%)		2 (2.1%)		
ACE inhibitors	26 (28.9%)		17 (18.3%)		
AT2 receptor blocker	23 (25.6%)		29 (31.2%)		
Beta blocker	36 (40.0%)		38 (40.9%)		
Antithrombotic drug	24 (26.7%)		25 (26.9%)		
DOAC	13 (14.4%)		17 (18.3%)		
Corticosteroids	11 (12.2%)		10 (10.8%)		
Immunosuppressive Drugs	4 (4.4%)		6 (6.5%)		
Nosocomial infection	8 (8.9%)		11 (11.7%)		

Data are median (Interquartile range) or n (%).

Significant values are in [bold].

*ICU* Intensive care unit, *ECMO* extracorporeal membrane oxygenation, *NIV* Non-invasive ventilation, *COPD* Chronic obstructive pulmonary disease, *ACE* Angiotensin-converting enzyme, *AT2* Angiotensin II, *DOAC* Direct oral anticoagulants.

Overall, the incidence of venous thromboembolic complications was high, affecting 46% (n = 84) of all patients. VTE manifested as deep vein thrombosis in 32% (n = 58), pulmonary embolism (PE) in 31% (n = 57) and catheter associated thrombosis in 3% (n = 5). Arterial thromboembolic events (myocardial infarction, stroke, systemic embolism or acute arterial thrombosis in peripheral or mesenterial arteries) affected 6% (n = 11).

Notable, 92 patients (50%) presented treatment-worthy bacteremia in blood culture next to sepsis. Septic shock at ICU admission was significantly more frequent in non-Survivors (19.4% vs 6.7%, Table 5). Besides, deceased patients showed amongst others higher need of additional supportive treatment of RRT, iNO and ECMO (Table 5). Non-survivors presented significantly higher rates of pleural effusion with the need of drainage (53% vs 36%, Table 6).

**Table 5:** ICU characteristics survival.

	Survivors	Range	Non-survivors	Range	*p*
n	90		94		
Intubated at ICU admission	30 (33.3%)		21 (22.3%)		
ARDS mild at ICU admission	7 (7.8%)		7 (7.4%)		
ARDS moderate at ICU admission	41 (45.6%)		44 (46.8%)		
ARDS severe at ICU admission	39 (43.3%)		43 (45.7%)		
Septic shock at ICU admission	6 (6.7%)		18 (19.4%)		**< 0.05**
First Horovitz-index at ICU	112.5 (83; 165)	52.5–450	105 (75; 142.5)	45–262.5	
Lowest Horovitz-index at ICU	75 (52.5; 90)	22.5–225	52.5 (45; 67.5)	22.5–135	
P_mean_ at admission [mbar]	19 (16; 22)	7–28	20 (18; 22)	8–30	
PEEP at admission [mbar]	13 (12; 15)	6–20	14 (12; 15)	6–20	
pH at admission	7.40 (7.36; 7.46)	7.17–7.62	7.37 (7.31; 7.42)	6.81–7.59	
PaCO_2_ at admission [kPa]	6.29 (5.38; 6.82)	3.23–9.86	6.64 (5.83; 7.52)	4.42–15.90	
SpO_2_ at admission [%]	94 (91; 96)	56–100	93 (89; 96)	64–100	
SOFA score at ICU admission	11 (8; 13)	5–16	12 (11; 14)	4–19	**< 0.05**
D-dimers at ICU admission [ng/ml]	4000 (1808; 7638)	484–20,000	6128 (4114; 10,994)	495–20,000	**< 0.05**
Lactate at ICU admission [mmol/L]	1.10 (0.85; 1.40)	0.40–3.30	1.30 (0.90; 1.90)	0.50–9.90	**< 0.05**
Duration mechanical ventilation ICU [days]	10 (6; 17)	2–56	13 (8; 17)	1–61	
Reintubation	2 (2.2%)		2 (2.1%)		
Prone position	48 (53.3%)		65 (69.1%)		**< 0.05**
Cycles of prone position	3 (2; 4)	1–14	4 (3; 7)	1–11	**< 0.05**
Tracheostomy	41 (45.6%)		41 (43.6%)		
Days from intubation to Tracheostomy	13 (10; 15)	3–26	11 (8; 15	3–21	
CRRT	15 (16.7%)		52 (55.3%)		**< 0.05**
Duration CRRT [hours}	337.01 (100.66; 483.67)	17.33–788.74	138.10 (30.17; 239.08)	1.44–906.53	**< 0.05**
ECMO	14 (15.6%)		35 (37.2%)		**< 0.05**
Duration ECMO [hours]	312.34 (208.30; 479.50)	70.78–1068.31	253.80 (163.27; 347.93)	16.78–577.63	
Cytosorb	0		8 (8.5%)		**< 0.05**
Duration Cytosorb [hours]			20.00 (17.17; 21.15)	8.67–51.00	
Red Cell Transfusion	5 (1; 8)	1–36	7 (3; 13)	1–40	
NO inhalation	12 (13.3%)		50 (53.2%)		**< 0.05**
Corticosteroid	73 (81.1%)		92 (97.9%		**< 0.05**
Immunoglobulin	2 (2.2%)		2 (2.1%)		
Convalescent plasma	20 (22.2%)		14 (14.9%)		
Remdesivir	14 (15.6%)		6 (6.4%)		**< 0.05**
Anticoagulation	90 (100%)		94 (100%)		
Argatroban at any time on ICU	8 (8.9%)		7 (7.4%)		**< 0.05**
UFH at any time on ICU	51 (56.7%)		89 (94.7%)		**< 0.05**
LMWH at any time on ICU	72 (80.0%)		43 (45.7%)		**< 0.05**
Bacteremia	36 (40.0%)		56 (59.6%)		**< 0.05**
Staph. aureus bacteremia	3 (3.3%)		9 (9.6%)		
Catheter associated bacteremia	12 (13.3%)		12 (12.8%)		
Antibiotics	84 (93.3%)		92 (97.9%)		
Antimycotics	12 (13.3%)		16 (17.0%)		
CRP maximum value [mg/l]	229.9 (144.4; 302.2)	31.4–584.7	305.5 (231.2; 373.5)	81.8–618.0	**< 0.05**
Interleukin 6 maximum value [pg/mL]	152.0 (80.5; 398.0)	8.6–21,728.0	674 (254; 2345)	15.9–792,732.0	**< 0.05**
Leucocytes maximum value [GPt/L]	17.19 (13.11; 22.43)	7.06–63.87	20.82 (16.75; 27.17)	3.14–63.64	**< 0.05**
Leucocytes minimum value [GPt/L]	7.22 (5.00; 9.39)	0.51–15.84	7.20 (4.63; 9.74)	0.20–22.47	
Procalcitonin [ng/ml]	1.31 (0.43; 6.02)	0.09–373.20	7.39 (2.20; 15.60)	0.15–148.40	**< 0.05**
Prothrombin fragment F1 + 2 [pmol/l]	393 (231; 780)	98.0–4948	541 (339; 1001)	73–4948	
Platelets maximum value [GPt/L]	355 (284; 461)	103–989	286 (219; 357)	48–617	**< 0.05**
Platelets minimum value [GPt/L]	170 (110; 219)	4–469	96 (47; 139)	1–414	**< 0.05**

Data are median (Interquartile range) or n (%).

Significant values are in [bold].

*ICU* Intensive care unit, *ARDS* Acute respiratory distress syndrome, *P*_*mean*_ Mean pressure, *PEEP* Positive end-expiratory pressure, *PaCO*_*2*_ partial pressure of carbon dioxide, *SpO*_*2*_ Oxygen saturation, *SOFA* Sequential organ failure assessment, *CRRT* Continuous renal replacement therapy, *NO* Nitric oxide, *UFH* Unfractionated heparin, *LMWH* Low-molecular-weight heparin, *Staph.* Staphylococcus, *CRP* C-reactive protein, *ECMO* extracorporeal membrane oxygenation.

**Table 6:** ICU outcome survival.

	Survivors	Non-survivors	*p*
n	90	94	
Duration of hospital stay [days]	19 (14; 27)	19 (14; 29)	
Duration of ANE-ICU stay [days]	14 (8; 19)	13 (9; 18)	
Duration of stay at UKD [days]	22 (15; 30)	14 (9; 20)	
VTE during ICU stay	37 (41.1%)	48 (51.1%)	
DVT	24 (26.7%)	34 (36.2%)	
Catheter associated thrombosis	2 (2.2%)	3 (3.2%)	
PE	25 (27.8%)	32 (34.0%)	
VTE before ICU admission	8 (8.9%)	9 (9.6%)	
Pneumothorax	8 (8.9%)	14 (15.1%)	
Lung emphysema	2 (2.2%)	7 (7.5%)	
Mediastinal emphysema	3 (3.3%)	10 (10.8%)	
Subcutaneous emphysema	6 (6.7%)	11 (11.8%)	
Pleural effusion	32 (35.6%)	49 (52.7%)	**< 0.05**
Fusion in lung	2 (2.2%)	13 (13.8%)	

Data are median (Interquartile range) or n (%).

Significant values are in [bold].

*ICU* Intensive care unit, *ANE-ICU* Intensive care unit of the Department of Anesthesiology and Critical Care Medicine, *UKD* University hospital Dresden, *DVT* Deep vein thrombosis, *VTE* Thromboembolic complications, *PE* Pulmonary embolism.

**Figure 2:**
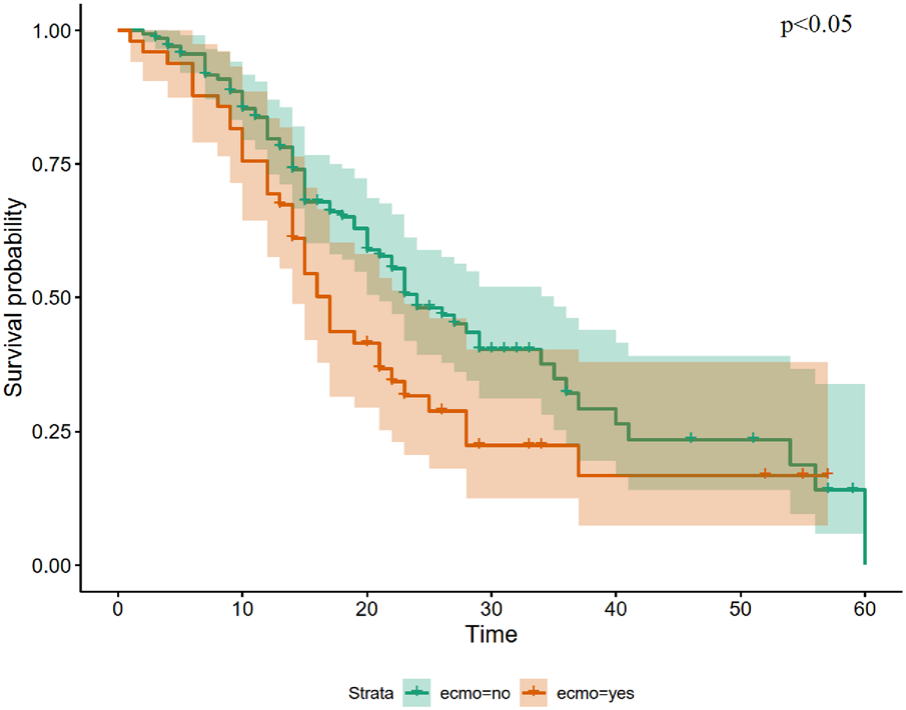
Kaplan–Meier Curves comparing ECMO therapy for COVID-19 ARDS. *ARDS* acute respiratory distress syndrome, *ECMO* extracorporeal membrane oxygenation. Time is indicated in days. Group comparison were performed using Log Rank test.

The estimated probability of 30 days survival in patients with the need of ECMO therapy was 22% (SE 6.7%) and worse than in patients without ECMO-therapy with 40% (SE 5.3%, p < 0.05, Fig. 2).

#### Risk factors associated with in-hospital mortality in regression analysis

In bivariate regression analysis the following variables were associated with higher in-hospital mortality: higher age, septic shock at ICU admission, higher SOFA score at ICU admission, d-dimer greater than 4000 ng/ml at ICU admission, invasive ventilation at ICU admission, need of RRT during ICU stay, need of inhaled nitric oxide therapy, need of ECMO therapy, lowest paO_2_ during ICU stay, maximum IL-6 values greater than 150 pg/ml during ICU stay, maximum PCT greater than 2 ng/ml during ICU stay, maximum values of platelets lower than 350 GPt/l, lowest value of platelets lower than 100 GPt/l and not conducting tracheotomy during ICU stay (Table 7).

**Table 7:** Cox regressions for hospital mortality.

Variable	Bivariate regressions		Known at admission		Full model	
	HR	CI	HR	CI	HR	CI
n	184		184		184	
Age	1.042**	1.020–1.064	1.051	1.023–1.080	1.092	1.053–1.132
Male	1.454	0.916–2.306	1.490	0.917–2.422	1.411	0.795–2.501
BMI: 35–40 kg/m^2^	0.884	0.492–1.586	1.178	0.630–2.206	1.922	0.961–3.843
BMI: > 40 kg/m^2^	1.084	0.499–2.353	1.718	0.715–4.128	3.380*	1.085–10.533
CCI	1.035	0.958–1.118	0.949	0.852–1.058	0.915	0.809–1.034
Septic shock at ICU admission	1.891*	1.145–3.121	1.258	0.728–2.174	1.692	0.906–3.161
SOFA score at ICU admission	1.125**	1.055–1.201	1.129**	1.037–1.230	1.084	0.957–1.227
D-Dimers at ICU admission: > 4000 ng/ml	1.524	0.997–2.331	1.621*	1.011–2.599	0.919	0.515–1.639
Logarithm of first Horovitz-index at ICU	0.901	0.580–1.399	0.949	0.573–1.571	1.369	0.741–2.530
Direct transfer to our ICU from other hospital	1.393	0.931–2.084	1.166	0.708–1.920	1.248	0.732–2.130
Intubated at ICU admission	1.652*	1.054–2.589	0.836	0.445–1.570	0.879	0.403–1.917
Time from first symptom to admission to our ICU	1.015	0.996–1.036	1.009	0.985–1.034	1.008	0.983–1.034
ECMO	1.542*	1.032–2.303			2.268*	1.193–4.311
CRRT	1.864**	1.274–2.726			1.216	0.684–2.162
NO inhalation	2.086**	1.425–3.055			2.434**	1.422–4.165
Prone position	1.071	0.714–1.605			1.108	0.641–1.917
PE	1.212	0.813–1.806			0.832	0.477–1.449
Pneumothorax	0.837	0.481–1.457			0.465	0.187–1.161
Lung emphysema	1.042	0.630–1.725			2.411	0.881–6.600
Mediastinal emphysema	1.009	0.534–1.904			0.896	0.247–3.247
Pleural effusion	1.215	0.830–1.776			0.915	0.561–1.492
Bacteremia	1.141	0.776–1.677			0.645	0.375–1.109
Logarithm of lowest Horovitz-index at ICU	0.357**	0.199–0.640			0.414*	0.189–0.907
Leucocytes maximum value: > 20 GPt/l	1.393	0.952–2.036			0.805	0.485–1.337
Interleukin 6 maximum value: > 150 pg/ml	2.272**	1.335–3.869			2.115	0.914–4.893
PCT maximum value: > 2 ng/ml	2.290**	1.462–3.588			1.832	0.938–3.577
CRP maximum value: > 400 mg/l	1.435	0.746–2.760			0.394	0.138–1.123
CRP maximum value: 200–400 mg/l	1.685	0.978–2.902			0.508	0.220–1.178
Platelets maximum value: > 350 GPt/l	0.488**	0.320–0.746			0.541*	0.302–0.969
Platelets minimum value: < 100 GPt/l	1.661**	1.135–2.430			0.921	0.512–1.657
Fusion in lung	1.383	0.767–2.494			0.871	0.430–1.766
Mycosis	1.079	0.714–1.631			1.071	0.626–1.832
Catheter associated bacteremia	0.753	0.439–1.291			0.703	0.354–1.397
Tracheostomy	0.660*	0.449–0.970			0.402**	0.243–0.664
DVT	1.184	0.796–1.760			1.253	0.730–2.152

Hazard ratios with 95%-confidence intervals for hospital mortality from bivariate Cox regression, Cox regression including covariates known at admission and Cox regression including all covariates (full model) (significance levels: * = 5%, ** = 1%).

*BMI* Body-Mass-Index, *CCI* Charlson ComorbidityIndex, *CI* Confidence interval, *CRP* C-reactive protein, *CRRT* Continuous renal replacement therapy, *DVT* Deep vein thrombosis, *ECMO* extracorporeal membrane oxygenation, *HR* Hazard ration, *ICU* Intensive care unit, *NO* Nitric oxide, *PCT* Procalcitonin, *PE* Pulmonary embolism.

Taking only variables into account, which were known at ICU admission, d-dimers > 4000 ng/ml (HR 1.641, CI 1.641–2.633), higher values of SOFA score (HR 1.129, CI 1.037–1.230) and higher age (HR 1.051, CI 1.023–1.080) showed the highest predictive value for in-hospital mortality (Table 7).

In multivariate full model regression analysis, morbid obesity with BMI > 40 kg/m^2^ was the strongest predictor for in-hospital mortality (HR 3.147, CI 1.000–9.897). Furthermore, higher age, need of inhaled nitric oxide therapy, need of ECMO therapy, maximum values of platelets lower than 350 GPt/l, lowest paO_2_ during ICU stay and not performing tracheotomy were associated with higher in-hospital mortality (Table 7).

### Midterm outcome and the prevalence of long-COVID

#### Characteristics of the cohort

Midterm follow-up was complete for 88% (n = 67) of the 76 patients discharged alive from hospital. Nine patients (12%) were lost during follow-up. At time of the telephone follow-up, 83% (56) of patients were alive and 55 patients participated in the survey, whereas 11 patients died during midterm follow-up. The midterm follow-up intervals varied from 8 to 20 months with median 11 months (IQR 10–11). The estimated probability of 8 months survival (midterm survival) after SARS-CoV-2 ARDS was 32.8% (SE 3.6%) in our cohort.

Of the survivors, 78% (n = 43) reported symptoms of Long-COVID associated with discomfort. The most common symptoms were fatigue (70%), shortness of breath (57%), impaired mental concentration (50%) and limb or muscle pain (50%). Long-COVID symptoms lead to hospital admission in 37% of all patients. Permanent home oxygen support was necessary in 11% and 6% remained on renal replacement therapy. Additionally, 15% needed outpatient care and 26% stayed in nursing homes or other comparable institutions.

Following discharge from our hospital, all patients were treated in rehabilitation institutions with a median stay of 56 days (range 14–246, IQR 28–98). The majority of our patients (n = 33; 60%) was already retired at the time of SARS-CoV-2 ARDS, but reintegration into work life was successful in 50% of all patients working before ICU stay (n = 11). The median EQ-VAS was 60 points (range 0–100; IQR 45–75).

#### Risk factors for developing long-COVID

In multivariate analysis for patients discharged alive from hospital, only obesity was associated with increased probability of developing Long-COVID symptoms. Thereby, the relative risk was higher in patients with BMI > 40 kg/m^2^ (RR 1.61, CI 1.26–2.06) than in patients with BMI between 35 and 40 kg/m^2^ (RR 1.37, CI 1.04–1.79, Table 8).

**Table 8:** Relative risk regressions for Long-COVID.

Variable	Bivariate regressions		Adjusted for age and sex	
	RR	CI	RR	CI
n	55		55	
Age	0.99	0.98–1.01		
Male	1.11	0.80–1.56		
BMI: 35–40 kg/m^2^	1.32	1.00–1.76	1.37*	1.04–1.79
BMI: > 40 kg/m^2^	1.56**	1.25–1.95	1.61**	1.26–2.06
CCI	1.01	0.95–1.08	1.03	0.96–1.11
Septic shock at ICU admission	1.14	0.71–1.83	1.14	0.72–1.82
SOFA-Score at ICU admission	0.98	0.93–1.04	0.98	0.93–1.04
ECMO	1.17	0.84–1.62	1.14	0.79–1.64
CRRT	1.09	0.73–1.63	1.08	0.70–1.66
Logarithm of lowest Horovitz-index at ICU	1.04	0.75–1.44	1.06	0.74–1.53
Logarithm of duration of mechanical ventilation at ICU	1.11	0.90–1.37	1.09	0.87–1.36
VTE during ICU stay	1.11	0.83–1.48	1.13	0.84–1.54
Direct transfer to our ICU from other hospital	1.03	0.77–1.38	1.06	0.80–1.40
DVT	0.99	0.72–1.36	1.00	0.72–1.38

Relative risks with 95%-confidence intervals for Long-COVID from robust Poisson regressions (significance levels: * = 5%, ** = 1%).

*BMI* Body-Mass-Index, *CCI* Charlson ComorbidityIndex, *CI* Confidence interval, *CRRT* Continuous renal replacement therapy, *DVT* Deep vein thrombosis, *ECMO* extracorporeal membrane oxygenation, *ICU* Intensive care unit, *RR* Relative risk, *VTE* Thromboembolic complications.

## Discussion

This study reported short-term and mid-term outcome of cARDS patients with the need of invasive ventilation and specialized ICU treatment and provided new insights in an area where data are still scarce.

### Short-term outcome

Data on short-term outcome for hospitalized COVID-19 patients as well as patients on ICU have been widely reported and large cohort studies are available, demonstrating hospital mortalities ranging from 42%^6^ to 73.7%^7^. However, COVID-19 can lead to ARDS making invasive ventilation and in severe cases ECMO support necessary^4,41,42^. In this context, the reported ICU mortality of 51% and in-hospital mortality of 59% in our ARDS cohort falls into the lower range of expectations, especially since we are a referral center where often the most critically ill patients are transferred from community hospitals. This referral bias limits our data to more severe ARDS cases and patients with non-invasive ventilation are not represented in this study. At the same time, this selection pattern puts our mortality rate into a favorable perspective, which is also demonstrated by a median initial SOFA score of 12 points at ICU admission, already predicting mortality rates up to 95%^43–45^. Other studies reported far different results for hospital mortality, mostly dependent on the number of invasively ventilated patients or the severity of ARDS. The more severe ARDS patients were included in the study, the higher the number of reported deaths leading to ICU mortality up to 84.6%^5^ and 85.7% for ECMO patients^46^.

Aim of this analysis was also to identify risk factors for inferior outcome. Our study suggests, that in particular BMI > 40 kg/m^2^ and the amount of d-dimers at ICU admission could be used to identify patients at increased risk for unfavorable outcomes close to admission. Of note, both parameters could causally be connected, since patients with increased BMI have been demonstrated to present with higher levels of plasminogen activator inhibitor 1 (PAI-1). Visceral fat has been reported to be the main physiological storage for PAI-1^47^ and higher PAI-1 values have been shown in obese patients. PAI-1 is released from infected, activated endothelial cells, adipocytes and platelets in septic patients^48^ and high PAI-1 levels are associated with worse outcome in COVID-19 patients^49^. PAI-1, emitted by monocytes, is a strong inhibitor of fibrinolysis^50^. Ranucci et al. showed that COVID-19 patients with worse outcome had up to sixfold higher PAI-1 levels compared to survivors^49^. In consequence of high plasma levels of PAI-1, fibrinolysis mediated by tissue plasminogen activator (tPA) and urokinase plasminogen-activator (uPA) may be severely reduced^51^ and could lead to a fibrinolytic shutdown, which is frequently seen in COVID-19 patients^52–55^. This could also explain why many of the critically ill COVID-19 patients are obese, or vice versa, why many obese patients develop more severe stages of COVID-19. It should be noteworthy, that BMI > 40 kg/m^2^ was shown as a strong risk factor for in-hospital mortality as well as the prevalence of Long-COVID symptoms. Similar results were found in a series of 3615 patients with COVID-19 from New York, US, those under 60 years of age with a BMI ranging from 30 to 34 kg/m^2^ had a 1.8-fold increase in the probability of ICU admission compared to patients with a BMI < 30 kg/m^2^. This likelihood increased to 3.6-fold among patients with a BMI ≥ 35 kg/m^2^^33^. Moreover, COVID-19 patients in ICUs had higher BMI than non-ICU patients (BMI, median 30.5 kg/m^2^ vs 28.77 kg/m^2^^56^. Furthermore, Salinas-Aguirre et al. reported an 1.88 fold increased mortality in patients with obesity > 30 kg/m^2^, investigating on 17.479 patients from Mexico^57^. A meta-analysis published by Yang et al. showed, that obesity > 30 kg/m^2^ is associated with increased risk of hospitalization, admission to ICU, need for invasive mechanical ventilation and mortality among COVID-19 patients^58^.

However, the only risk associated with the development of Long-COVID was obesity with BMI > 40 kg/m^2^ (RR 1.61, CI 1.26–2.06). While some studies likewise suggest obesity to be a possible risk for the development of post-COVID^59^, female sex is mentioned more often as a risk factor for the development of post-COVID^60,61^, which could not be confirmed in our study.

Complications during ICU stay were high in survivors and non-survivors. The occurrence of thromboembolic complications was up to 50% in our cohort but had no significant influence on patient’s outcome. This is surprising compared to other studies^17^. We can only hypothesize, that our consistent screening at ICU admission helped to early identify patients with ATE/VTE and subsequent increased anticoagulation therapy protected from inferior outcome. Noteworthy, the high VTE rates observed in our and many other COVID studies are not caused by ARDS itself, since VTE rates in patients with severe influenza ARDS were demonstrated to be considerably lower at 3%^62^.

As one would expect, patients in our cohort with inhaled nitric oxide therapy (iNO) and/or ECMO-therapy showed significant worse outcomes. Concomitantly, this subgroup showed higher SOFA-score and lower Horovitz-indices. Additional to MV and prone position iNO was regularly applied for treatment of severe hypoxemia in ARDS patients preliminary or instead (in cases, considered unsuitable for) of ECMO support. According to current recommendations, ECMO support is suggested as rescue therapy^38,63^. Complications related to ECMO therapy and mortality remain high^3,41^. Recent studies reported mortality for COVID-19 patients after ECMO support ranging from 22% in a very small cohort (9 patients) from Zurich^64^ up to 86% in other small series (7 patients) from Munich^46^ and 39% in the preliminary data from the ELSO-registry study^42^. A recent germanwide study did not recommend liberally ECMO use in COVID-19 ARDS (cARDS) patients and summarizes that the unconditional use of ECMO therapy in COVID-19 must be carefully considered and advanced age should be considered as a relative contraindication^65^. Indication for ECMO support should be critically discussed for every individual patient, considering structural lung damage, comorbidities, multi-organ failure and acceptable potential patients’ outcome. Taking the high number of critical ill patients into account, the limited number of available ECMO-devices, there could be an additional bias towards more conservative decision making.

Bacteremia and sepsis in the course of COVID-19 infection were frequent in our cohort, requiring antibiotic therapy necessary in 95% of all cases. However, proof of bacteremia was only possible in 50%. The other patients received calculated antibiotic therapy considering impaired organ function accompanied by elevated inflammatory parameters, e.g. procalcitonin. Another recently published study highlights the importance for IL-6 and PCT measurement as predictive biomarkers for COVID-19 severity^66^. Septic shock was treated in our department in accordance to national guidelines^37^, with fluid and catecholamine support as well as renal replacement therapy in case of acute kidney injury KDIGO stage 3^67^, metabolic acidosis, hyperkalemia or volume overload. Special approaches, like clearing inflammatory cytokines with CytoSorb filters, were only used in a small number of patients as a rescue therapy because of lack of evidence^68^, especially in patient with cARDS^67,69,70^. Hospital mortality in our patients who presented with septic shock exceeded the one reported in Non-COVID patients (40–60%)^71^.

### Midterm outcome and the prevalence of long-COVID

In addition to in-hospital outcomes, we reported mid-term outcomes of our ARDS patients after a minimum of eight months after hospital discharge. 83% of all patients (56/67) discharged from hospital were alive. Considering the whole cohort, this results in a probability of 8 months survival after admission to ICU for cARDS limited to 32.8%, which highlights the life threatening severity of COVID-19. Additional, 78% of our patients with available midterm follow-up reported symptoms of Long-COVID with median EQ-VAS of only 60 points.

A similar study from Spain showed a 5.2% mortality (5 out of 97 patients) 6 months after ICU release. The study was performed using data from 7 different ICUs^72^. Of the 92 surviving patients 91 were interviewed regarding their life-quality following the EQ-5D-3L. 61 (67%) patients reported a decreased quality of life, most commonly impeded were mobility (56%), pain (48%) and anxiety or depression (46%)^72^. Likhvantsev et al. reported 16 (7.2%) patients deceased out of 222 patients discharged from ICU^73^ although as many as 34 patients were lost to follow-up. Of the 125 patients which completed the survey, 68% reported serious problems regarding physical health while 48% reported serious problems regarding mental health^73^. Another recently published study including 41 patients with an average ICU stay of only 8.42 days concentrates on the psychological impairments. 12.2% had moderate depression, 2.4% severe depression. 14.6% of patients suffered from mild to moderate anxiety, 12.2% severe anxiety. 29.3% reported acute PTSD^74^.

In summary, short and midterm outcome of patients with COVID-19 developing severe ARDS was not satisfying. The high prevalence of Long-COVID shows the long healing path of severe COVID-19 ARDS patients, which goes far beyond the discharge from hospital. Obesity seems to be a serious risk factor associated with increased in-hospital mortality and the occurrence of Long-COVID.

### Study limitations

As this is a retrospective study, it faces all the limitations associated with this type of analyses. We have observed different variations in patient characteristics and quantities that are likely to influence the prognosis. The main bias in this study is the inhomogeneous disease stage, caused by a high number of patients admitted from other hospitals or ICUs. Despite the fact that some statistics must be interpreted with caution, the key findings of this study reflect our clinical observations. Therapeutic approaches changed during the time period, some medications, e.g. corticoids, became standard treatment, while others could not reach significant improving effect in recently published studies and were not further used.

## Conclusion

ARDS in COVID-19 patients is characterized by high morbidity and mortality. Complications during ICU stay are frequent. Midterm survival was acceptable with > 80%, but most of the patients developed Long-COVID symptoms associated with discomfort. To identify patients at high risk, laboratory parameters for inflammation and d-dimers can be helpful. Especially patients with BMI > 40 kg/m^2^ are at high risk for inferior short-term outcome and prevalence of Long-COVID.

## Data Availability

The datasets are not publicly available due to data sharing protocols but are available from the corresponding author on reasonable request.

## Abbreviations

aPTT: Activated partial thromboplastin time
ARDS: Acute respiratory distress syndrome
ATE/VTE: Thromboembolic complications
AWMF: Association of the Scientific Medical Societies in Germany
aXa: Anti-Xa activity
BMI: Body-Mass-Index
cARDS: COVID-19 associated acute respiratory distress syndrome
CAT: Catheter associated thrombosis
CCI: Charlson ComorbidityIndex
cCUS: Complete compression ultrasound
CI: Confidence interval
COVID-19: Coronavirus disease 2019
CRP: C-reactive protein
CTPA: Computed tomography pulmonary angiography
DIC: Disseminated intravascular coagulation
DVT: Deep vein thrombosis
ECMO: Extracorporeal membrane oxygenation
EDTA: Ethylene diamine tetraacetic acid
e.g.: Exempli gratia
ELSO: European extracorporeal life support organization
EOLIA: ECMO to rescue acute lung injury in severe ARDS
EQ-VAS: EuroQol visual analogue scale
HIT: Heparin-induced-thrombocytopenia
HR: Hazard ratio
ICU: Intensive care unit
IL: Interleukin
IQR: Interquartile range
iNO: Inhaled nitric oxide
INR: International normalized ratio
KDIGO: Kidney Disease: Improving Global Outcomes
MV: Mechanical ventilation
NICE: National Institute for Health and Care Excellence
NIV: Non-invasive ventilation
PaO_2_: Partial pressure of oxygen
PCT: Procalcitonin
PE: Pulmonary embolism
PEEP: Positive end-expiratory pressure
PF 1 + 2: Prothrombin fragment 1 + 2
P/F ratio: Horovitz-index
PT: Prothrombin time
PTSD: Post-traumatic stress disorder
RR: Relative risk
SARS-CoV-2: Severe acute respiratory syndrome coronavirus 2
SE: Standard error
SOFA: Sequential organ failure assessment
SOP: Standard operating procedure
VET: Viscoelastic testing
VT: Venous thrombosis
VTE: Venous thromboembolism
WHO: World Health Organization
